# Identification of a novel mutation in *PATL2* gene associated with the germinal vesicle arrest of oocytes

**DOI:** 10.1016/j.bbrep.2024.101886

**Published:** 2024-11-22

**Authors:** Aili Yu, Zhiqing Huang, Hang Shi, Yanying Lin, Xuefen Cai, Zhanghong Ke, Beihong Zheng, Yan Sun

**Affiliations:** a Center of Reproductive Medicine, Fujian Maternity and Child Health Hospital, Fuzhou, 350001, China; b College of Clinical Medicine for Obstetrics & Gynecology and Pediatrics, Fujian Medical University, Fuzhou, 350001, China; c Fujian Maternal-Fetal Clinical Medicine Research Center, Fuzhou, 350001, China

**Keywords:** Assisted reproductive technology, Oocyte maturation abnormality, Germinal vesicle arrest, *PATL2*

## Abstract

The aim of this study was to investigate the genetic factors of a patient with germinal vesicle arrest in oocytes. Clinical data and blood samples were collected from the patient and some were amplified for high-throughput gene and Sanger sequencing. Several molecular and cellular experiments were performed to determine the association between the gene mutation and germinal vesicle arrest. Two mutation sites (c.1282G > T and c.1247C > A) were found in the *PATL2* gene. The c.1282G > T mutation is associated with oocyte maturation abnormalities according to previous research, whereas c.1247C > A is a novel mutation of unknown clinical significance. In cell transfection experiments, qRT-PCR, immunofluorescence, and Western blotting revealed that the mRNA and protein levels of the *PATL2* gene with the c.1247C > A mutation were reduced. Sanger sequencing suggested that the patient inherited the *PATL2* mutations from her parents via a compound heterozygous mode of inheritance. Collectively, this study describes the *PATL2*-gene c.1247C > A mutation associated with germinal vesicle arrest in oocytes, providing a useful target for genetic and background tests for patients presenting with oocyte maturation abnormalities and/or germinal vesicle arrest following multiple unsuccessful attempts with assisted reproductive technology.

## Highlights

• GV arrest of oocytes can be caused by genetic mutations.
• The c.1247C > A of *PATL2* gene was first reported in this study.
• The c.1247C > A of *PATL2* gene is proved associated with the GV arrest in oocytes.

## 1 Introduction

There are three crucial regulatory points in human oocyte development: prophase of meiosis I (MI), metaphase of MI, and metaphase of meiosis II (MII). Normally, human oocyte development is arrested in the prophase of MI and completes MI at puberty under the influence of sex hormones. After expulsion of the first polar body, the oocyte remains in MII metaphase and undergoes arrest until successful fertilisation, at which time the second polar body is expelled, resulting in the formation of a zygote and, eventually, embryo [1]. The availability of normal mature oocytes is an important determinant of human reproductivity, with 8.6%–15 % of female patients suffering from infertility associated with the production of at least one type of immature oocyte [2]. In a minority of patients undergoing assisted reproductive technology (ART), most or all oocytes obtained through controlled ovarian hyperstimulation are immature [3], a phenomenon known as oocyte maturation abnormality (OMA). Patients with OMA often suffer from primary infertility and fail to produce enough high-quality embryos or achieve desirable clinical pregnancy outcomes even after multiple ART treatments.

OMAs have been classified into four types: germinal vesicle (GV), metaphase I (MI), metaphase II (MII), and mixed arrests [4]. Oocyte maturation is a relatively complex process and is regulated by many molecules. Various studies have described the pathologic mechanisms of GV and MI arrest. GV arrest is thought to occur due to the failure of maturation-promoting factor activation, as evidenced by the GV-stage-arrest of surgically isolated oocytes, showing low rates of maturation and fertilisation and impaired embryonic development potential. Several studies have demonstrated that GV arrest can be caused by genetic mutations [5]. A 2017 study showed that the protein associated with the topoisomerase II homolog 2 (*PATL2*) gene is associated with GV arrest, which was further supported by related case reports [6,7].

This study describes the molecular characteristics of an OMA in a female patient with primary infertility that underwent two unsuccessful ART treatments. We identified two mutations (including a novel mutation) in the *PATL2* gene through high-throughput gene sequencing, and explored the relationship between *PATL2* gene mutation and OMAs.

## 2 Materials and methods

### 2.1 Patient

This study describes the genetic characteristics of a patient presenting with primary infertility at the Reproductive Center of Fujian Provincial Maternity and Children's Hospital (Fuzhou, China) in 2018 who underwent two cycles of ART treatment for tubal infertility. The specific characteristics of the patient are summarized in Table ST1 (Supplementary Table ST1). The study was approved by the Institutional Review Board of Fujian Provincial Maternity and Children's Hospital (2021KR037), and written informed consent was obtained from the patient.

### 2.2 Evaluation of oocyte maturation and embryonic development

Morphological assessments of oocyte maturation, fertilisation, and embryonic development were performed using light microscopy. MII oocytes were associated with polar body (PB) 1 expulsion (Supplementary Figure SF1). Oocytes with a complete GV were considered to be at the GV stage; oocytes containing no germinal vesicle or polar body, at metaphase I (MI); and oocytes with the first polar body extruded, at MII. The GV and MI oocytes were considered immature. Fertilisation was assessed at 17 ± 1 h after intracytoplasmic sperm injection (ICSI). The resultant embryos were cultured until Day 5 or 6.

### 2.3 Detection of mutation sites

The patient was tested for gene mutations associated with OMAs using high-throughput gene sequencing.

Sanger sequencing and validation: 2 mL of peripheral blood was collected from the patient and her parents in EDTA anticoagulant tubes. Genomic DNA was extracted using a QIAamp DNA kit (Qiagen, Hilden, Germany). All the coding regions of the *PATL2* gene were amplified. The PCR primers are shown in Table ST2 (Supplementary Table ST2).

Evaluation of variant pathogenicity: The frequencies of mutant alleles were searched using the ExAC browser and Genome Aggregation Database (gnomAD). Homologous and heterologous sequences of the human PATL2 protein were aligned using MultAlin software. The scale-invariant feature transform (SIFT) algorithm was used to evaluate the PATL2-related protein variants and pathogenicity. Splice-site mutations were evaluated using the NNsplice database.

### 2.4 Cell culture, plasmid construction and cell transfection

Human embryonic kidney 293T derivative 17 (HEK 293T/17) cells were collected from the Fujian Medical University Union Hospital Institute of Haematology (Fuzhou, China). The HEK 293T/17 cells were cultured in high-sugar DMEM containing 10 % PAN Biotech foetal bovine serum and a 1 % penicillin–streptomycin antibiotic mixture at 37 °C and 5 % CO_2_.

Construction of lentiviral overexpression vectors: The PATL2 fragment and c.1247C > A-variant PATL2 fragment were synthesised by BioSune (Shanghai, China). Gene fragments were cloned into the pCDH-His plasmid (SBI, CD513B-1, System Biosciences) by double digestion (FastDigest *Nhe*I, FastDigest *Bam*HI) to obtain the plasmids pCDH-PATL2-His (**PATL2**-WT) and pCDH-PATL2-c.1247C > A-His (**PATL2**-Mutation).

Cell transfection: HEK 293T cells (5 × 10^6^) were plated on 15 cm cell culture dishes and transfected proportionally with lentiviral helper and pCDH plasmids (pCMV-VSV-G:psPAX2:pCDH = 1:2:3) to obtain **PATL2**-WT and **PATL2**-mutation plasmids. An empty plasmid was transfected into HEK 293T cells as a negative control (293T-WT).

### 2.5 Total RNA extraction and RT-qPCR

Total RNA was extracted using Trizol reagent (Tiangen, Beijing, China). Single-stranded cDNA was reverse transcribed using 1 μg of total RNA. The samples were loaded analysed on a Step OnePlus Real-Time PCR system (Applied Biosystems, Foster City, CA, USA), and the relative expression of target genes was calculated using *β-actin* as an internal reference. The primer sequences are shown in Table ST3 (Supplementary Table ST3).

### 2.6 Immunofluorescence analysis

The cells were fixed on a slide with 4 % paraformaldehyde, repeatedly immersed and washed, permeabilised with 0.5 % Triton X-100 (dissolved in PBS), washed again and dried, and blocked by dropwise addition of goat serum at room temperature. The slides were then placed in a wet box, treated with PATL2 primary antibody (1:200), and incubated at 4 °C overnight. After repeated immersion and washing, a fluorescent secondary antibody (1:1000) was added dropwise to the slides, which were thereafter incubated at 25 °C. DAPI was added dropwise for nuclear staining, following incubation in the dark. Finally, the slides were mounted with an anti-fade mounting medium and imaged using a confocal fluorescence microscope.

### 2.7 Western blotting

The cells were collected and total protein was extracted on ice with RIPA lysis buffer (Solarbio, Beijing, China) containing protease inhibitors (cOmplete, Roche, Switzerland). Thereafter, 40 μg of sample protein was collected for SDS-PAGE. Blotted membranes were incubated with a rabbit anti-His-Tag monoclonal antibody (6X His tag, 1:1,000, Abcam) followed by sheep anti-rabbit secondary antibody (1:10,000, Boster Bio, Wuhan, China). Autoradiography was performed using an ECL luminescence reagent (Merck Millipore, Burlington, MA, USA) and gel documentation system to obtain images of the Western blots. GAPDH (Cell Signalling Technology, Danvers, MA, USA) was used as internal reference. Grayscale values were calculated using ImageJ software.

### 2.8 Statistical analysis

Data are presented as the mean ± standard error of the mean (SEM). Student's *t*-test (GraphPad Prism) was used for comparisons between groups. Differences with *p* < 0.05 were considered statistically significant. The following labels in the figures indicate the level of significance: ∗*p* < 0.05, ∗∗*p* < 0.01, ∗∗∗*p* < 0.001.

## 3 Results

### 3.1 Morphological abnormalities in oocytes

In the couple with primary infertility, the female partner showed normal menstruation, ovarian function, and sex hormone levels and no obvious organic pathology of the uterus or adnexa; the male partner suffered from oligospermia and teratospermia. After two cycles of ovulation induction with different protocols, 11 and 6 oocytes were obtained, respectively (Supplementary Table ST1), all of which were arrested in the GV phase (Supplementary Figure SF1).

### 3.2 PATL2 *gene mutations in patient*

High-throughput gene sequencing identified two heterozygous mutations in the *PATL2* gene, namely c.1282G > T, p.E428X (chr15:44960623C > A) and c.1247C > A, p.P416H (chr15:44960658G > T) (Fig. 1). Gene database and literature searches revealed that the former is a pathogenic variant. We found no record of the latter and classified it as a variant of uncertain significance (searches and variant pathogenicity results are shown in Table 1). The amino acid alteration was found to be conserved among mammals (Supplementary Figure SF2). Sanger sequencing revealed that the patient's father was heterozygous for the c.1247C > A mutation and her mother was heterozygous for the c.1282G > T mutation (Fig. 1), confirming that these mutations inherited from her parents via a compound heterozygous mode.

**Fig. 1:**
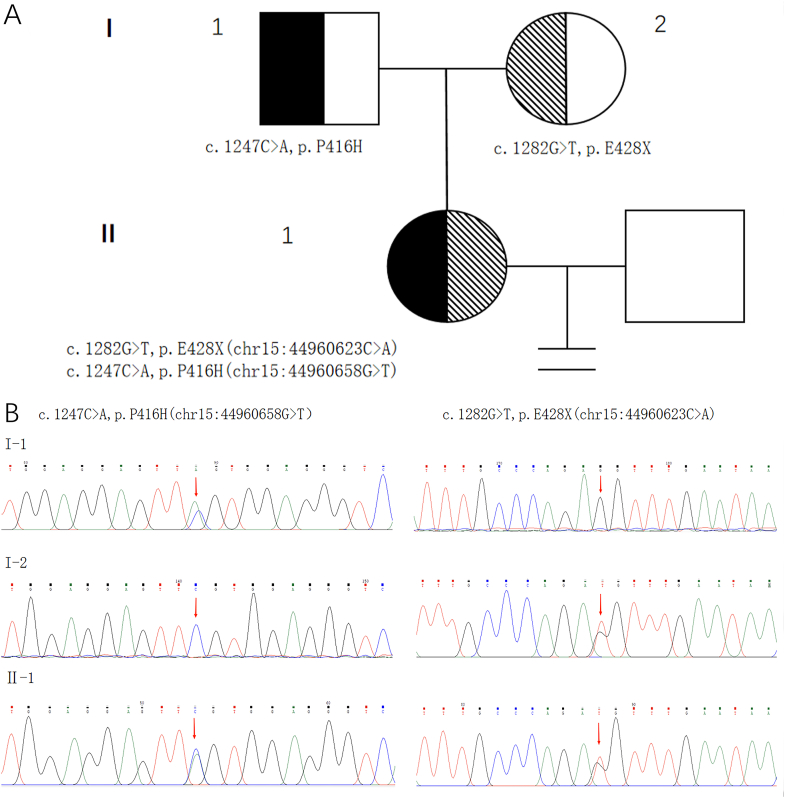
Pedigree and Sanger sequencing map of the patient A: Pedigree showing inheritance pattern of *PATL2* mutations in patient's family. The patient inherited the mutations from her parents, consistent with recessive Mendelian inheritance. I-1 is the patient's father, I-2 is the patient's mother, and II-1 is the patient. B: Validation of mutations by Sanger sequencing.

**Table 1:** Mutations in *PATL2* gene.

Nucleotide change	Amino acid change	Zygosity	Disease name	Mode of inheritance	Sequence variation classification
c.1282G > T	p.E428X	Heterozygous	OMA	AR	Pathogenic
c.1247C > A	p.P416H	Heterozygous	OMA	AR	Uncertain clinical significance

### 3.3 mRNA and protein expression of PATL2

We investigated the effects of the novel mutation (c.1247C > A) on *PATL2* gene expression in HEK 293T cells transfected with the lentiviral overexpression plasmids **PATL2**-WT and **PATL2**-mutation.

**Fig. 2:**
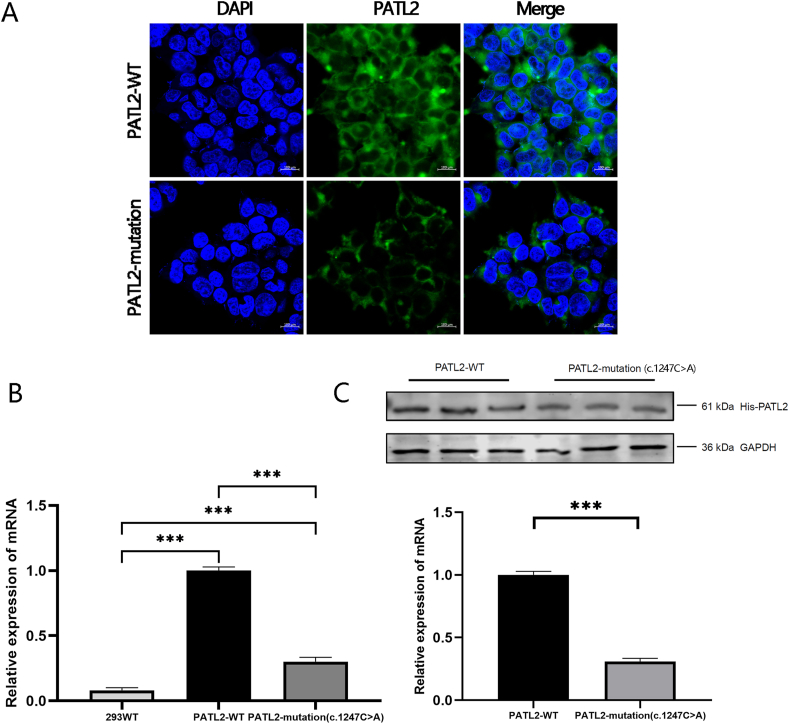
PATL2 expression after transfection of HEK 293T cells with **PATL2**-mutation (c.1247C > A) overexpression plasmid A: After HEK 293T cells were transfected with **PATL2**-WT or **PATL2**-mutation (c.1247C > A) plasmids, their nuclei were stained with DAPI and PATL2 expression was visualized with anti-PATL2 antibodies. The HEK 293T cells exhibited decreased PATL2 protein expression following transfection with the **PATL2**-mutation (c.1247C > A) plasmid. B: After transfection with the **PATL2**-mutation (c.1247C > A) plasmid, *PATL2* expression was decreased, whereas the wild-type HEK 293T cells did not exhibit any transcription of PATL2. C: After HEK 293T cells were transfected with **PATL2**-mutation (c.1247C > A) plasmids, PATL2 protein expression was decreased compared to that after **PATL2**-WT transfection.

Immunofluorescence analysis showed that the c.1247C > A mutation caused a significant decrease in *PATL2* gene expression in the cytoplasm of HEK 293T cells compared to the wild-type PATL2 (Fig. 2A). RT-qPCR showed that the **PATL2**-mutation transfection significantly downregulated the mRNA expression of *PATL2* compared to transfection with **PATL2**-WT (Fig. 2A). Western blot analysis showed that PATL2 protein expression was significantly lower following transfection with **PATL2**-mutation than that with **PATL2**-WT (Fig. 2A).

Previous studies have demonstrated an association between the *PATL2* gene and oocyte maturation. Our results suggested that the c.1247C > A mutation in the *PATL2* gene can affect its intracellular expression, thereby affecting oocyte maturation. Therefore, this mutation is likely associated with the GV arrest in oocytes.

## 4 Discussion

The global prevalence of OMAs is speculated to be around 0.1%–1% of all women, and its aetiology remains poorly understood. Our study suggests that the diagnosis of patients with multiple recurrent oocyte maturation failures would benefit from the analysis of genetic factors using whole-exome sequencing (WES)—a genomics method that captures and enriches DNA from exons across the whole genome for high-throughput sequencing. The efficiency and cost-effectiveness of WES in identifying the genetic variants that alter protein sequences have led to its widespread use in the study of pathogenic genes. It also shows great potential in the screening of mutant genes in patients with OMA. In a 2018 cohort study, Christou-Kent et al. used WES and Sanger sequencing to reveal that 26 % of patient with OMA were homozygous for a nonsense mutation in *PATL2* [8].

The expression and function of the *PATL2* gene and its homologs have been studied in *Xenopus laevis*, mice, and humans. The *PATL2* gene is relatively conserved across species but may vary in function. Among them, the *PATL2 Xenopus* homolog encodes an oocyte-specific single-stranded DNA binding protein that inhibits mRNA accumulation during follicular development. The overexpression of PATL2 leads to oocyte maturation arrest [9]. In mice, the PATL2 protein has no effect on the oocyte maturation process [10]. However, Patl2^−/−^ female mice exhibited subfertility, OMAs, and decreased embryonic development potential. Conversely, the human *PATL2* gene has a broad spectrum of effects on oocyte maturation [11]. Mutations in *PATL2* can result in GV stage arrest, MI arrest, or PB1 oocytes with large polar bodies, or a decrease in mature oocytes in rare cases. The specific function of *PATL2* in oocyte maturation remains unclear; however, the abundant expression of PATL2 was observed in human oocytes at the GV stage, and immunofluorescence experiments have shown that PATL2 protein expression in the cytoplasm starts in the primary follicle stage and clears during the GV stage [11]. Mutations in PATL2 can lead to the accumulation of missense PATL2 proteins or a dramatic decrease in PATL2 protein expression [12]; though, whether this is caused by failure of the normal initiation of meiosis in oocytes or is related to the phenotypic diversity of OMAs remains unclear.

OMAs are characterized by the recurrent production of mostly immature oocytes that cannot be cultured to maturity in vitro, failure of fertilization by ICSI, and an inability to form mature oocytes even with assisted in vitro maturation [13]. Patients with oocyte failure due to genetic mutations are unable to obtain healthy embryos through multiple ovulation inductions. Currently, the only effective ART strategy is egg donation. Gene editing with CRISPR/Cas9 technology has shown effective results in the treatment of OMAs caused by mutations in the *TUBB8* gene [14]; however, this method is technically demanding and ethically controversial. For OMAs caused by mutations in the *TRIP13* gene, oocyte maturation can be stimulated by injecting gene-specific cRNAs [15]. To develop more effective personalized treatment approaches, further research is required to determine whether mature oocytes can be obtained from the experimental injection of *PATL2* cRNA in patients with *PATL2* mutations and describe the functions and molecular mechanisms of *PATL2* in oocyte meiosis.

Our study identified the potential impact of the novel mutation c.1247C > A on the mRNA and protein expression of PATL2 in a patient presenting with abnormal oogenesis. Immunofluorescence and qRT-PCR analyses showed that the c.1247C > A mutation resulted in a significant downregulation in PATL2 protein and mRNA expression in HEK 293T cells (Fig. 2A and B). This suggests that the mutation affects the expression of PATL2 at the transcriptional level. Subsequent studies should consider the effect of this mutation on the post-transcriptional modifications of PATL2, such as m6A, acetylation, and glycosylation. 3D protein structure simulation (Supplementary Figure SF3) indicated that the c.1247C > A mutation alters the normal amino acid sequence of the PATL2 protein, further highlighting its potential effect on PATL2 protein expression and transcription, and disease development. In conclusion, the present study revealed that the novel mutation c.1247C > A is a major pathogenic factor in patients encountering GV arrest in oocytes, and may be a useful target for drug development. Meanwhile, our findings also suggest that early genetic testing for *PATL2* in patients with OMAs could promote early diagnosis and treatment for improved patient outcomes.

## CRediT authorship contribution statement

**Aili Yu:** Writing – original draft, Visualization, Supervision, Software, Methodology, Investigation, Funding acquisition. **Zhiqing Huang:** Writing – original draft, Visualization, Supervision, Software, Methodology, Investigation, Funding acquisition. **Hang Shi:** Writing – review & editing, Writing – original draft, Validation, Resources. **Yanying Lin:** Validation, Methodology, Formal analysis. **Xuefen Cai:** Visualization, Data curation, Conceptualization. **Zhanghong Ke:** Software, Resources, Investigation. **Beihong Zheng:** Methodology, Investigation. **Yan Sun:** Methodology, Investigation.

## Funding information

The project was financially supported by Fujian Provincial Natural Science Foundation of China (2020J05279 and 2023J011229), Fujian Provincial Natural Science Foundation of China (2019-2-23), Joint Funds for the Innovation of Science and Technology, Fujian province (2020Y9140), Major Scientific Research Program for Young and Middle-aged Health Professionals of Fujian Province (2022ZQNZD010), Doctoral Research Initiation Fund of Fujian Maternity and Child Health Hospital (YCXB 18-04), Fujian Maternity and Child Health Hospital Science Startup Foundation (YCXZ 19–27), and Innovation Platform Project of Science and Technology, Fujian province (2021Y2012).

## Declaration of competing interest

The authors declare that they have no known competing financial interests or personal relationships that could have appeared to influence the work reported in this paper.

## Figures and Tables

**figs1:**
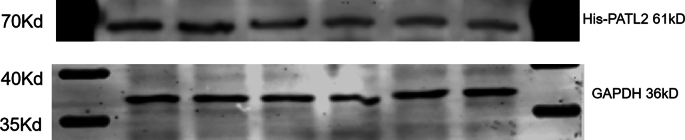


## Data Availability

Data will be made available on request.
