# Supplementary material for: Identification of a novel mutation in PATL2 gene associated with the germinal vesicle arrest of oocytes
Source: Biochem Biophys Rep. 2024 Nov 22;40:101886. doi: 10.1016/j.bbrep.2024.101886 (PMC11625204; doi:10.1016/j.bbrep.2024.101886)
Supplement: Multimedia component 1 [file mmc1.docx]

**Supplementary material**

Table ST1 Baseline clinical characteristics of the patient

| Cycle | Age | Duration of  infertility | BMI | b-FSH  mIU/mL | LH  mIU/mL | E2  pg/mL | AMH  ng/mL | Protocol | Oocytes obtained | GV | MI | MII | Fertilised |
| --- | --- | --- | --- | --- | --- | --- | --- | --- | --- | --- | --- | --- | --- |
| 1 | 36 | 10 | 21.83 | 5.83 | 1.8 | 45 | 2.83 | GnRH-α prolonged | 11 | 11 | 0 | 0 | 0 |
| 2 | 37 | 11 | 21.83 | 8.33 | 2.4 | 41 | 2.80 | PPOS | 6 | 6 | 0 | 0 | 0 |

Table ST2 PCR primers of *PATL2* gene for Sanger sequencing

| Primer name | Primer sequence (5′→3′) |
| --- | --- |
| PATL2-0104-F | CTGATTCTGAAGCACCACGG |
| PATL2-0104-R | GGGGAGGTGAAGTTGTAGTGG |

Table ST3 Target gene primer sequences

| Primer name | Sequence (5′→3′) |
| --- | --- |
| CCDC69-F | GGTGGAGGAGTCATTCTGGA |
| CDC69-R | TGGAGGTCCTCATTTTCCTG |
| Eef1e1-F | TGCAGAAGAAAAAGCAATCG |
| Eef1e1-R | ACCTCTGTGTGCCTCAGCTT |
| Prr11-F | GTTCTGATCACCCTGGAGA |
| Prr11-R | AGCGAGACTGGGTTTCTGA |
| SKa2-F | GCTGGACCAGAGTCGAGTTC |
| SKa2-R | TCGCCGCAGTTTTCTCTTCTT |
| PATL2-F | CACTTGCCTCCTTGCATTTTC |
| PATL2-R | CTGGGTCAGATGACTGAACCT |


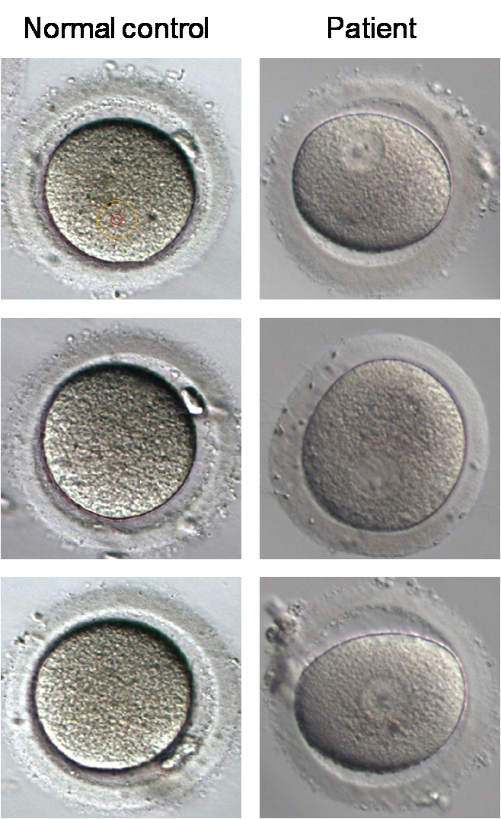


Figure SF1 Comparison of oocyte morphology between the normal control oocytes and oocytes from the patient (400× magnification).


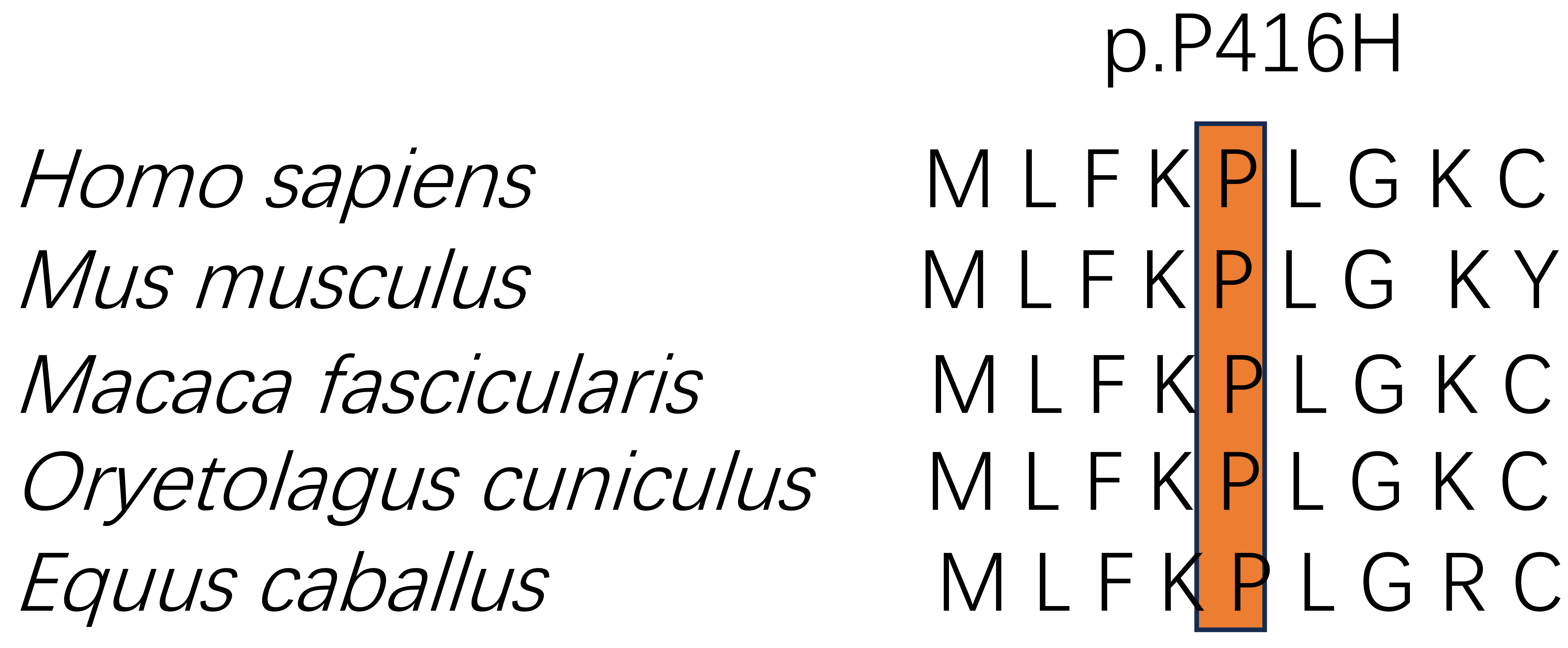


Figure SF2 Conservation analysis of altered amino acids among five mammalian species.


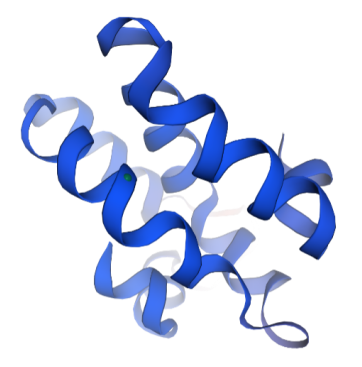


Figure SF3 Structure of the PATL2 protein and its amino acid sequence. The green label indicates the location of the c.1247C>A mutation affecting the normal expression of the proline residue at this site (amino acid sequence: FLQVLSVRKGKALVARLLPFLPQDQAVTILLAITHHLPLLVRRDVADQALQMLFKPLGKCISHLTLHELLQGLQGLTLLPPGSSERPVTVVLQNQFGISL).
